# The significance of cerebrospinal fluid dynamics in adolescent idiopathic scoliosis using time-SLIP MRI

**DOI:** 10.1038/s41598-024-63135-3

**Published:** 2024-05-28

**Authors:** Yusuke Tomita, Mitsuru Yagi, Fumiko Seki, Yuji Komaki, Satoshi Suzuki, Kota Watanabe, Morio Matsumoto, Masaya Nakamura

**Affiliations:** 1 Department of Orthopaedic Surgery, Keio University School of Medicine, Tokyo, Japan; 2 Department of Orthopaedic Surgery, School of Medicine, International University of Health and Welfare, 852 Hatakeda Narita, Chiba, 286-0124 Japan; 3 Department of Physiology, Keio University School of Medicine, Tokyo, Japan; 4 Live Animal Imaging Center, Central Institute for Experimental Animals, Kanagawa, Japan

**Keywords:** Time-SLIP MRI, Cerebrospinal fluid, Adolescent idiopathic scoliosis, Neuroscience, Diseases, Medical research

## Abstract

Adolescent idiopathic scoliosis (AIS) affects approximately 3% of the global population. Recent studies have drawn attention to abnormalities in the dynamics of the CSF as potential contributors. This research aims to employ the Time-Spatial Labeling Inversion Pulse (Time-SLIP) MRI to assess and analyze cerebrospinal fluid (CSF) dynamics in AIS patients. 101 AIS patients underwent Time-SLIP MRI. Images were taken at the mid-cervical and craniocervical junction regions. The sum of the maximum movement distances of CSF on the ventral and dorsal sides of the spinal canal within a single timeframe was defined and measured as Travel Distance (TD). Correlations between TD, age, Cobb angle, and Risser grade were analyzed. TD comparisons were made across Lenke classifications. TD for all patients was a weak correlation with the Cobb angle (r = − 0.16). Comparing TD between Lenke type 1 and 5, type 5 patients display significantly shorter TD (p < 0.05). In Risser5 patients with Lenke type 5 showed a significant negative correlation between Cobb angle and TD (r = − 0.44). Lenke type 5 patients had significantly shorter CSF TD compared to type1, correlating with worsening Cobb angles. Further analysis and exploration are required to understand the mechanism of onset and progression.

## Introduction

Adolescent idiopathic scoliosis (AIS) is the most common form of spinal curvature seen in children aged 10 to 18^1,2^. While the exact etiology of AIS remains elusive, it represents a multifactorial disease with genetic, neuromuscular, biomechanical, and other factors implicated in its onset and progression^2^. One area of intrigue within this multifaceted disease panorama has been the potential role of cerebrospinal fluid (CSF) dynamics in AIS patients^3–5^. The CSF, encasing the brain and spinal cord, plays a pivotal role in supporting neural structures, providing nutrients, and clearing metabolic waste^3,4^. Any perturbations in its flow dynamics could potentially influence the surrounding neural and osseous tissues, including the vertebral column. Recently, animal studies, particularly those employing zebrafish models, have been instrumental in uncovering the potential link between CSF dynamics and scoliosis^4,6^.

Grimes et al. utilized a zebrafish model of idiopathic scoliosis to highlight how defects in ependymal cell cilia development and consequent disruptions in CSF flow could lead to spinal deformities^6^. This underscores the importance of cilia-driven CSF flow during spine morphogenesis, and even more intriguingly, the restoration of cilia motility post scoliosis onset halted the progression of the spinal curve.

Drawing from these animal models, the recurring theme is the centrality of CSF dynamics, whether through flow or its detection by specialized neurons, in maintaining normal spine morphology and preventing deformities. While zebrafish models provide initial insights into CSF dynamics and scoliosis, it is critical to consider the scale-dependent nature of fluid hydrodynamics when extrapolating these findings to humans. Several prior human studies have explored this connection, using modalities like contrast MRI^3,5,7,8^. These studies, along with certain animal models, have laid the groundwork suggesting that alterations in CSF dynamics might be linked to the development or progression of scoliosis. However, while promising, the usage of contrast MRI presents certain challenges. Not only is it invasive, posing potential risks to patients, but the labelling time of CSF during the study is inherently limited^3,5,7,8^. This confines our understanding and ability to capture the complete picture of CSF dynamics over extended durations.

The advent of Time-Spatial Labeling Inversion Pulse magnetic resonance imaging (Time-SLIP MRI) has provided a potential solution to this limitation^9^. The Time-SLIP MRI is an MRI technique that visualizes CSF flow in the brain and spinal cord by using a selective inversion recovery pulse to "label" specific regions, tracking their signal intensity differences over time^9^. This method, which doesn't require external contrast agents, is adept at revealing CSF flow dynamics and is instrumental in diagnosing conditions like normal pressure hydrocephalus, Chiari malformation, and syringomyelia. Its ability to capture slow-moving or pulsatile fluid motion offers distinct advantages in assessing neurological conditions. Taking into account these discoveries and the promise of Time-SLIP MRI, our study aims to investigate the involvement of CSF dynamics in AIS.

## Materials and methods

This study was approved by the institutional review board at our institutions (Keio University School of Medicine Ethics Committee), and all subjects consented and agreed with their inclusion. All methods were performed in accordance with the relevant guidelines and regulations. Written informed consent was obtained from all patients & their parents for publication of this article.

### Patient enrolment

We prospectively enrolled patients with AIS from a single center.

### Inclusion and exclusion criteria

#### Inclusion criteria

Patients eligible for this study must have:

1. Patient with verified idiopathic scoliosis diagnosed between 10–18 years of age.
2. Undergone Time-SLIP MRI.
3. Completed standing posteroanterior (P/A) and lateral whole spine radiographs.

#### Exclusion criteria

Patients were excluded from the study if:

1. They had a pathological condition such as congenital anomalies, neuromuscular disorders, pre-existing neural axis abnormality or any syndromic conditions known to be associated with skeletal deformities.
2. MRI findings indicated specific anomalies, such as Chiari type 1 malformation and associated syringomyelia.

Notably, two patients were excluded due to the presence of these conditions in their MRI results.

### Data collection and radiographic assessment

The demographic and clinical data collected included the age at diagnosis, age at index surgery, sex, and comorbidities. Full-length standing whole-spine radiographs obtained at baseline were analyzed. Radiographic data included the following measurements: Risser grade, Cobb angle of main thoracic curve and lumbar curve.

### MRI acquisition

The temporal resolution of our Time-SLIP MRI scans was 12,000–16,000 ms, with each time-resolved image scan lasting approximately 1800 ms. This detailed specification is crucial for understanding the dynamics captured in each movie included in the manuscript, ensuring clarity in the temporal aspects of CSF flow measurement.

*Patient positioning* Patients were instructed to assume a supine position with a neutral cervical alignment throughout the examination.

### MRI equipment and settings:

- *Machine:* 1.5 T and 3 T MR imager (Vantage Elan and Vantage Galan 3 T, Canon Medical Systems, Tochigi, Japan).
- *Time-SLIP imaging parameters:*
  - 2D single-shot T2-weighted imaging
  - Pulse-wave-gated preparation scan
  - TR: 12,000–16,000 ms (depends on pulse rate), TE: 80 ms
  - Echo train spacing: 5 ms
  - Flip/Flop angle: 90°/160°
  - Field of view: 260 × 260 mm
  - Matrix size: 256 × 256
  - Slice thickness: 5 mm
  - Pixel bandwidth: 651 Hz
  - Inversion Time: 1800–3700 (increases at intervals of 100 ms)

*Time-SLIP MRI* After the initial scan, a Time-SLIP MRI was conducted for comparison. The imaging primarily captured the sagittal plane, concentrating on the mid-cervical and craniocervical junction regions.

*Respiratory control* Prior to undergoing TIME-SLIP MRI scans, all participants were given standardized instructions aimed at minimizing respiratory impacts on CSF dynamics measurements^10^. They were directed to maintain slow and regular breathing patterns throughout the scan, avoiding both breath-holding and deep breathing. Along with standard breathing instructions, participants were guided to follow synchronized breathing patterns structured into intervals of 2, 4, 6, 8, and 10 s. To further reduce the impact of respiratory variations, respiratory gating technology was employed during the MRI scans. Imaging sequences were initiated at the start of each exhalation phase, aligning with the most stable respiratory phase to minimize motion artifacts. This method ensures consistent capture of CSF dynamics during the part of the respiratory cycle with the least variation, substantially improving the reliability of TD measurements.

### Image analysis and quantitative evaluation

**Figure 1:**
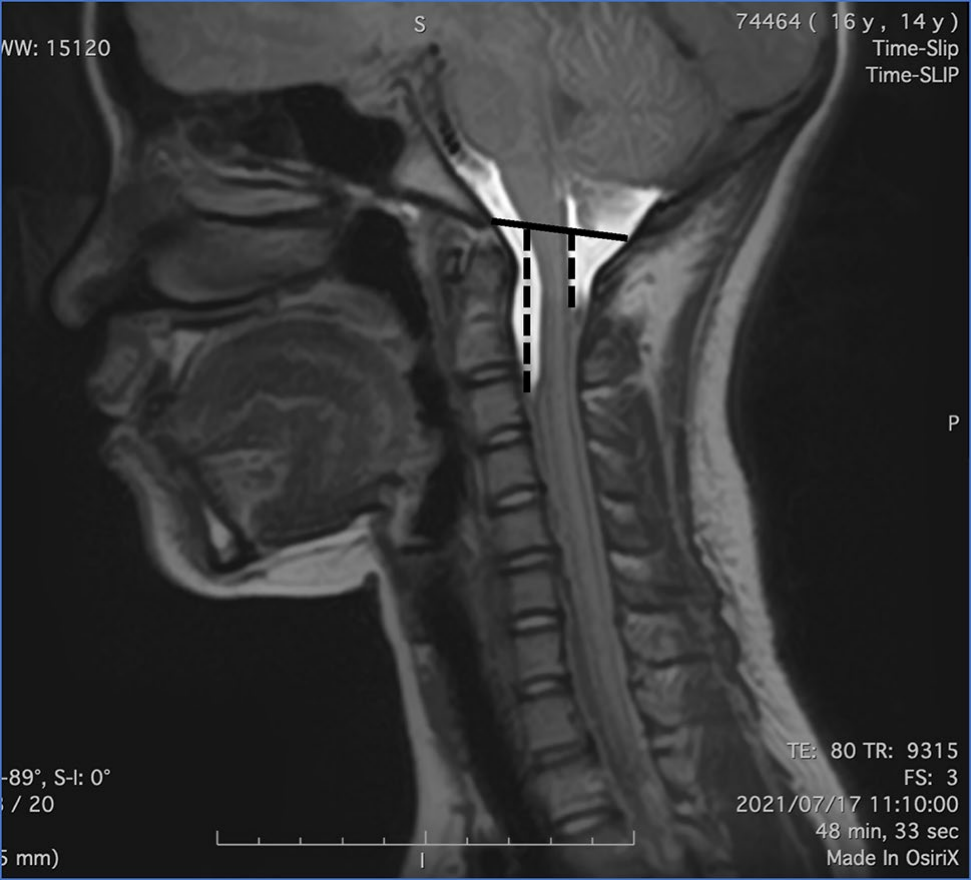
Travel distance measurement using Time-SLIP MRI. The solid line indicates the Bastion-opisthion line. The dotted lines indicate the maximum movement distances of CSF on ventral and dorsal side of the spinal canal. Travel distance was measured as the sum of the maximum movement distances of CSF on the ventral and dorsal sides of the spinal canal from Bastion-opisthion line within a single timeframe was defined and measured as TD.

Following acquisition, all MR images transferred to an independent workstation, which was equipped for multiplanar reformatting (MPR) of source images and maximum intensity projections (MIPs). A quantitative measure was also employed, calculating the Travel Distance (TD) of the CSF on the Time-SLIP MRI. The sum of the maximum movement distances of CSF on the ventral and dorsal sides of the spinal canal from Bastion-opisthion line within a single timeframe was defined and measured as TD (Fig. 1). Two board certified orthopedic surgeons independently measured all radiograph parameters. One examiner measured once, and the other examiner measured twice a week apart after brief lecture for standardize the measurement. Examiners were blinded to patient clinical information and other measurement. Correlations between TD, age, Cobb angle, and Risser grade were analyzed. TD comparisons were made across Lenke classifications^11^.

### Statistical analysis

The intraclass correlation coefficient (ICC) of the intra- and inter-observer reliabilities of measurements obtained from Time-SLIP MRI images of 10 randomly selected patients were calculated. We classified the ICC values according to the criteria introduced by Aubin et al. ; < 0.24, 0.25–0.49, 0.50–0.69, 0.70–0.89, and 0.90–1.0 were considered to be poor, low, fair to moderate, good, good to excellent, respectively^12,13^. Differences for TD between Lenke type 1 and type 5 were compared by Student’s t test, and chi-square test where appropriate^12^. Correlation between TD and cobb angle of major curve, age, were analyzed by Correlation coefficient test. TD comparisons between Lenke curve types were analyzed using a Student’s t-test, confirming our initial assumption of normal distribution. Normality was verified through the Shapiro–Wilk test, ensuring the appropriateness of parametric testing in our analysis (Lenke type 1; W = 0.971, p = 0.188, Lenke type5; W = 0.980, p = 0.794).

. A p value less than 0.05 with a confidence interval (CI) of 95% was considered statistically significant. All analyses were performed using the Statistical Package for the Social Sciences (SPSS statistics version 28.0, IBM Corp., Armonk, NY).

### Ethical approval

This study was approved by the appropriate institutional review board.

## Results

### Characteristics of the patient cohort

Table 1 describes the demographic and clinical characteristics of the patient cohort. Among the participants, there were 6 males and 95 females, with an average age of 15.3 ± 2.8 years. The distribution of Lenke curve types showed a predominance of types 1 and types 5. Specifically, 56 patients were classified as Lenke type 1, 10 as type 2, 1 as type 4, 32 as type 5, and 2 as type 6. The average Cobb angle for the main curve across the cohort was measured to be 34.7 ± 12.2°.

**Table 1:** Description of patient cohort.

Variables	
Age (year-old)	15.3 ± 2.8
Gender	
Male	6 (5)
Female	95 (95)
Cobb angle (°)	34.7 ± 12.2
Lenke type	
Type 1	56 (55.4)
Type 2	10 (9.9)
Type 3	0 (0)
Type 4	1 (1.0)
Type 5	32 (31.7)
Type 6	2 (2.0)
Risser grade	
0	5 (5.0)
1	6 (5.9)
2	7 (6.9)
3	10 (9.9)
4	31 (30.7)
5	42 (41.6)

Means and standard deviations Percentage in parenthesis.

### Intra- and inter-observer reliabilities of TD measured by time-SLIP MRI

Table 2 presents the reliability of TD measurements from Time-SLIP MRI. Intra-observer ICC values were 0.989 [95% CI 0.957–0.997], indicating excellent consistency. Inter-observer ICCs, similarly, showed excellent reliability at 0.966 [95% CI 0.935–0.996]. These findings underscore the high reproducibility of TD measurements using Time-SLIP MRI.

**Table 2:** Intra- and inter-observer reliabilities of TD measured by time-SLIP MRI.

	Intraclass correlation coefficients	p value
Intra-rater reliability	0.989 [0.957–0.997]	p < 0.001
Inter-rater reliability	0.966 [0.935–0.996]	p < 0.001

Values are shown as ICC [95% confidence interval].

### Correlation between TD and Cobb angle *of major* curve

**Figure 2:**
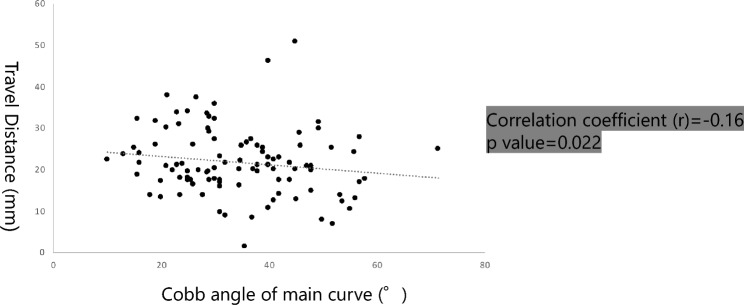
Correlation between the TD and Cobb angle of major curve. There was a weak negative correlation between the TD and the Cobb angle of major curve (r = − 0.16).

**Figure 3:**
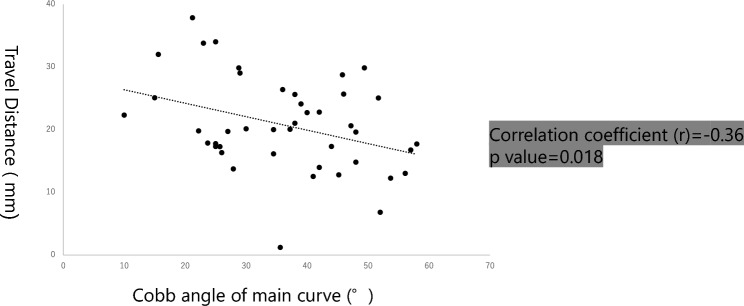
Correlation between the TD and Cobb angle of major curve in Risser 5 patients. The sub-analysis of skeletally mature patients revealed moderate correlation between TD and the Cobb angle of major curve (r = − 0.36).

The average TD for the entire cohort of patients was found to be 21.6 ± 8.0 mm. A poor negative correlation (r = − 0.16) was observed between TD and the Cobb angle of the major curve across all patients. This indicates that as the severity of the scoliotic deformity, reflected by larger Cobb angles, increased, there was a corresponding decrease in CSF flow, leading to shorter TDs (Fig. 2). Recognizing that the curve magnitude at skeletal maturity typically indicates the definitive curve magnitude, we specifically chose skeletally mature patients (Risser 5) for a more detailed sub-analysis. Within this mature subgroup, the correlation became more pronounced, with a correlation coefficient (r) of − 0.36, underscoring the observed trend between pronounced Cobb angles and decreased CSF flow, resulting in shorter TDs (Fig. 3).

### Comparison of TD between Lenke type 1 and Lenke type 5 patients

**Figure 4:**
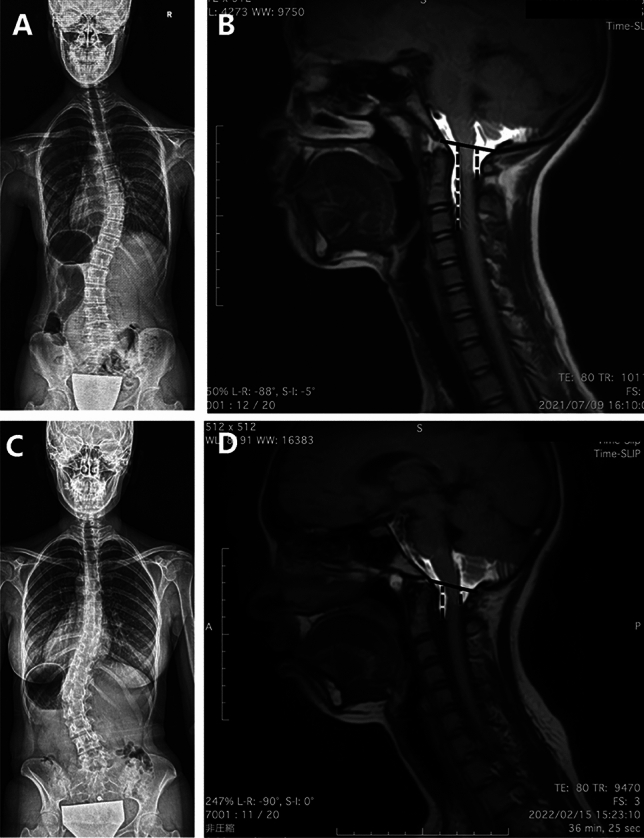
Representative Time-SLIP MRI images of Lenke type 1 and type 5 AIS patients. (**A**) Standing whole spine radiograph of 14-year-old female Lenke type 1 patient. Cobb angle of the major curve was 33 degrees and the Risser grade was 4. (**B**) Time-SLIP MRI of 14-year-old Lenke type 1 patient. The TD was 37.3 mm. (**C**) Standing whole spine radiograph of 19-year-old female Lenke type 5 patient. Cobb angle of the major curve was 39 degrees and the Risser grade was 5. (**D**) Time-SLIP MRI of 19-year-old Lenke type 5 patient. The TD was 6.3 mm.

When comparing TD between Lenke type 1 and Lenke type 5 patients, it was found that Lenke type 5 patients had a significantly shorter TD compared to Lenke type 1 patients (Lenke type 1 vs. Lenke type 5: 22.9 ± 7.7 mm vs 19.6 ± 7.0 mm, p = 0.046, Fig. 4, Supplemental Movies 1 and 2).

**Figure 5:**
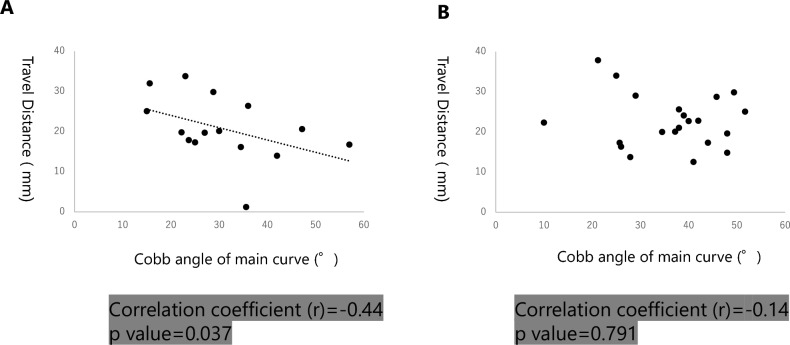
Correlation between the TD and Cobb angle of major curve in Lenke type 1 and Lenke type 5 patients. (**A**) Moderate correlation was observed between the Cobb angle and the TD in Lenke type 5 patients (r = − 0.44).The corresponding p-value for this correlation is 0.037. (**B**) No correlation was observed between the Cobb angle and the TD in Lenke type 1 patients.

Additionally, In the subgroup analysis of Risser 5 patients, a moderate negative correlation was observed in Lenke type 5 patients (r = − 0.44). The corresponding p-value for this correlation is 0.037, indicating statistical significance (Fig. 5A), while no correlation was observed in the Lenke type 1 patient cohort (Fig. 5B). Similarly, a shorter TD was noted in surgically treated Lenke type 5 patients, although it did not reach statistical significance, hence we operated (operative vs. non operative: 12.8 ± 8.4 mm vs. 22.7 ± 6.3 mm, p = 0.057).

## Discussion

### Reliabilities of TD measured by time-SLIP MRI

Reliability in medical imaging is pivotal for accurate diagnosis and treatment. The findings from our study on TD measurements using Time-SLIP MRI are promising. The ICCs showed that a single observer can consistently reproduce results across evaluations, with values nearing perfect reliability. The inter-observer ICCs also indicated excellent agreement between different observers, suggesting that Time-SLIP MRI images are clear and likely guided by a standardized measurement protocol.

### Comparison of CSF dynamics measurement techniques

There has been ongoing debate regarding the measurement procedures for assessing CSF dynamics. Various methodologies highlight different aspects of CSF flow, each with unique clinical implications.

Burman et al. measures the maximal volume of CSF displaced from the cranium to the spinal canal during each respiratory and cardiac cycle using phase-contrast MRI^14^. This method quantifies how physiological changes like breathing affect CSF flow, offering insights into CSF-to-blood toxin clearance and overall CSF circulation. It explores the influence of respiratory and cardiac rhythms on CSF dynamics, crucial for understanding toxin clearance and the mixing of cranial and spinal CSF. This approach is particularly relevant for studies on sleep, hydrocephalus, or evaluating therapeutic interventions targeting CSF circulation.

Inoue et al. integrates the total movement distance of CSF from the velocity–time graph across the entire CSF pulsation cycle^15^. This comprehensive method captures all movements of CSF, providing a holistic view of CSF flow characteristics throughout the cardiac cycle. It offers cumulative data beneficial for diagnosing and understanding conditions like Chiari Malformations, where generalized CSF flow disruption is involved. Our study focuses on the TD, specifically the peak extent of CSF displacement. This measurement offers insights into the potential maximum impact of CSF dynamics on surrounding neural and spinal structures. Our methodology aligns with previously reported measurements by Ohtonari et al. and Yamahata et al., who both have utilized same techniques to assess CSF dynamics in various pathological conditions^16,17^. This alignment underscores the validity and consistency of our approach within the broader research community. While our focus on TD provides a straightforward, clinically relevant measure of CSF displacement, Burman’s approach offers a broader perspective on how CSF volume is modulated by physiological cycles. Each method contributes uniquely to the understanding of CSF dynamics, and recognizing these differences is crucial for applying the appropriate technique to specific clinical or research questions. Our discussion emphasizes the distinct yet complementary nature of these methodologies in the assessment of CSF dynamics. By outlining the specific applications and advantages of each method, we aim to enhance the understanding and application of appropriate CSF measurement techniques in both clinical and research settings. The choice of method should be guided by the specific clinical requirements, the nature of the neurological condition being studied, and the specific CSF dynamics that need to be understood or monitored.

### Overall correlation of CSF flow and curve magnitude in AIS patient

This study elucidates a nuanced relationship between the dynamics of CSF, represented by TD, and the severity and type of curvature in AIS, quantified by the Cobb angle and Lenke type. A cohort of 101 AIS patients was examined, and a poor negative correlation was identified between TD and the Cobb angle of the major curve, hinting at an inverse relationship between CSF dynamics and curve severity.

The correlation becomes particularly pronounced in skeletally mature patients (Risser 5), implying that the relationship between CSF movement and curve progression may be more significant in patients who have attained skeletal maturity. The Cobb angle at this mature stage might symbolize the definitive final angle for the patient. This finding prompts further investigation into the developmental dynamics of AIS and the potential role of CSF dynamics in influencing the evolution of spinal curvature as patients age.

The findings presented add an insightful layer to the existing literature. Algin et al. conducted a pioneering study on the usage of 3 T PC-MRI and 3D-SPACE-VFAM for evaluating AIS^18^. They identified statistically significant differences between AIS patients and controls in peak velocity values and flow-void phenomenon scores, hinting at an association of AIS with CSF flow disturbances.

The utilization of MRI techniques, particularly 3D T2-weighted spin-echo MRI, for preoperative assessment in AIS patients, was also discussed by Duchaussoy et al.^5^ Their study emphasized the comparability and reliability of MRI against CT, suggesting MRI's potential as a safer, radiation-free alternative. Prior research has also probed the craniocervical junction and CSF flow dynamics in AIS or Chiari I malformations patients^7,8^. Chu et al. identified morphological distinctions such as low-lying cerebellar tonsils and expanded foramen magnum dimensions in AIS subjects^7^. Despite these variations, CSF velocities at the foramen magnum were not markedly distinct between AIS and controls. This is particularly intriguing in light of our findings, suggesting that while CSF velocities may not differ significantly, the TD could be indicative of underlying CSF flow disturbances, potentially linking to the progression or manifestation of scoliosis.

MRI with contrast has revolutionized the field of diagnostic imaging, offering unparalleled soft tissue visualization without ionizing radiation. However, there are several limitations associated with contrast-enhanced MRI, particularly when it comes to studies involving CSF dynamics or flow, mainly due to shorter labeling time and invasiveness^19^. Shorter labeling times can restrict the ability to visualize slower fluid movements and limit the temporal resolution of CSF dynamics. Additionally, the use of a contrast agent implies an invasive procedure^19^. While this is generally safe, there's always a risk associated with any invasive procedure, including allergic reactions or other adverse reactions such as Nephrogenic Systemic Fibrosis in pediatric patients.

In the context of CSF dynamics, these limitations can be especially pertinent, as a non-invasive, longer-duration labeling method like Time-SLIP can provide insights into fluid flow that are challenging to capture with conventional contrast MRI techniques. Notably, Time-SLIP MRI has been highlighted in multiple studies for its non-invasive evaluation of CSF dynamics. Takeuchi et al. applied Time-SLIP in a syringomyelia case, unveiling unique CSF flow patterns otherwise unseen with conventional imaging^20^. Ohtonari et al. further emphasized the utility of Time-SLIP MRI in assessing combined ventral and dorsal CSF dynamics at the craniocervical junction in Chiari malformation type I patients^16^. Their study posits the importance of understanding both ventral and dorsal CSF dynamics, emphasizing that this combined approach offers a more holistic view.

In our study, utilizing Time-SLIP MRI, which allows for an extended labeling duration, we identified a potential association between CSF dynamics, as evidenced by the TD measurements, and the severity of the scoliosis, indicated by the Cobb angle.

### Correlation between TD and Curve magnitude in Lenke type 5 AIS patient

Distinctive patterns emerged upon comparing AIS types based on the Lenke classification system. Lenke type 5 patients exhibited a notably shorter TD in contrast to those classified as Lenke type 1. This pronounced difference in TD between the two Lenke types could shed light on the diverse manifestations of AIS progression and varied responses to therapeutic interventions.

A more compelling aspect was the evident correlation between TD and the Cobb angle in skeletally mature Lenke type 5 patients. In comparison, this correlation was absent in Lenke type 1 patients. Such differences in correlation suggest that the mechanisms driving these two AIS types might be distinct. Recognizing these variances underscores the need for a nuanced, patient-centric approach tailored to each AIS type. Furthermore, the shorter TD observed among surgically treated Lenke type 5 patients, albeit not reaching statistical significance, raises intriguing questions about the potential influence of surgical procedures on CSF dynamics and its subsequent implications for AIS progression. Our surgical criteria predominantly catered to Lenke type 5 patients exhibiting a Cobb angle beyond 40 degrees upon reaching skeletal maturity or those anticipated to reach this threshold. The intriguing proximity of these findings to statistical significance highlights the need for further research, potentially steering enhanced surgical decision-making rooted in individual CSF dynamics.

While we don't have definitive answers explaining these disparities, past research provides some avenues for exploration^21,22^. In a clinical setting, Lenke types 1 (thoracic curve types) often present with narrower pedicle and a tendency for the enlarged spinal canal^21^. Abul-Kasim et al. detailed that Lenke type 1 patients exhibited unique pedicle width patterns with smaller, asymmetrical pedicles in comparison to individuals without scoliosis^21^. This difference was especially accentuated at the scoliotic apex. Wang et al., in their study on scoliosis patients associated with Chiari malformation/Syringomyelia, highlighted pedicle width differences, particularly around the apical region, that paralleled patterns noticed in AIS patients^22^. Yet, they did pinpoint certain deviations in thoracolumbar vertebrae. Algin et al. added another dimension to this discussion by pointing out that Lenke type 5 displayed a slower peak velocity for CSF compared to Lenke type 1 using 3 T PC-MRI and 3D-SPACE-VFAM^18^. Although this wasn't statistically significant, this observation might be attributed to the small sample size and the broad variation in velocity (with 14 patients for Lenke type 1 and only 5 for Lenke type 5).

As previously mentioned, Chu, et al. found no significant difference in the peak velocity of CSF at the craniocervical junction between AIS patients and healthy individuals^7^. However, they reported an enlargement of the anteroposterior diameter of the foramen magnum in AIS patients compared to healthy controls^7^. While their study did not perform a sub-analysis based on curve type, they concluded that the enlargement of the foramen magnum in AIS patients might be a compensatory response^7^. Drawing from this idea, we can propose a hypothesis: If abnormal CSF dynamics is one of the factors leading to AIS, then Types 1 and 2 might be compensating for this by enlarging their spinal canal and foramen magnum. On the other hand, in lumbar curve types like Lenke type 5, which are further from the craniocervical transition, this compensatory mechanism might not be in play. This could result in a shorter distance of CSF movement, potentially explaining why our study found a shorter TD in Lenke type 5.

Collectively, these studies underscore that diverse spinal morphology across scoliosis types might play pivotal roles in influencing CSF dynamics on the onset/progression of scoliosis, or conversely, the onset/progression of scoliosis may influence CSF dynamics.

### Comparative analysis of CSF dynamics across different studies

Our study utilized the TD measurement to assess CSF dynamics in AIS patients, drawing upon methodologies similar to those reported by Ohtonari et al. and Yamahata et al.^16,17^ These researchers provided foundational insights into the impact of spinal pathologies on CSF movement, which directly informed our approach.

Yamahata et al. investigated CSF dynamics at the craniovertebral junction (CVJ) in patients with cervical spinal canal stenosis using Time-SLIP MRI^17^. They reported significantly reduced Total Length of Motion (LOM) in patients with severe stenosis compared to controls, highlighting how spondylotic changes can restrict CSF flow. The mean total LOM for their control group was 16.0 ± 8.4 mm, which serves as a comparative benchmark for normal CSF dynamics. Ohtonari et al. explored CSF dynamics in patients with Chiari Malformation Type I, using Time-SLIP MRI to measure the combined ventral and dorsal CSF dynamics at the CCJ^16^. They found significant differences in total LOM between patients requiring craniocervical decompression and those who did not, underscoring the clinical relevance of assessing CSF dynamics in spinal pathologies.

While our study focuses on AIS patients and employs similar imaging techniques, the populations and specific conditions investigated differ from those in the aforementioned studies. Nevertheless, the general principles and outcomes regarding CSF dynamics provide a useful context. Our findings suggest altered CSF dynamics in Lenke type 5 patients, much like the alterations observed by Yamahata et al. in severe cervical canal stenosis^17^. Although direct comparisons are challenging due to differences in patient populations and conditions, the trends observed across these studies emphasize the potential neurological impacts of altered spinal anatomies on CSF flow. The insights gained from these comparative studies highlight the importance of precise CSF dynamics measurement in diagnosing and managing conditions associated with altered CSF flow. In AIS, understanding the maximum extent of CSF movement may help predict areas at risk during spinal curvature correction. This is analogous to the role of CSF dynamics in determining the necessity for surgical interventions in conditions like Chiari Malformation as demonstrated by Ohtonari et al.^16^.

### Study limitations

Several limitations must be acknowledged to contextualize our findings within the broader scope of Adolescent Idiopathic Scoliosis (AIS) research:

First, the number of participants included in our study was limited, potentially reducing the generalizability of our findings to the broader AIS population. Larger, multi-center studies could help confirm our preliminary findings and enhance the robustness of the data. Second, despite the use of advanced imaging techniques such as Time-SLIP MRI, there are inherent limitations associated with this technology that could affect the accuracy or resolution of our measurements. Integrating Time-SLIP MRI with other advanced imaging modalities might offer a more comprehensive understanding of CSF dynamics and their potential role in scoliosis progression.

Third, the cross-sectional design of our study constrains our ability to deduce causal relationships between CSF dynamics and the severity of scoliosis. Although our study identifies associations between CSF dynamics and AIS, it is crucial to emphasize that cross-sectional designs cannot establish causality. Longitudinal studies are necessary to determine whether changes in CSF dynamics directly contribute to the progression of scoliosis. Our findings suggest a potential role for CSF dynamics in influencing scoliosis progression, but definitive conclusions cannot be drawn at this stage. Notably, patients with Lenke type 5 AIS, even those requiring surgery, exhibited abnormal CSF dynamics, suggesting an association that merits further investigation, especially in the context of pronounced Cobb angles. Forth, The lack of a control cohort comprising healthy adolescents is a significant limitation, stemming primarily from the ethical and logistical challenges associated with planning invasive examinations for this population. Future non-invasive or minimally invasive techniques may allow for the inclusion of control groups, providing a baseline for comparing CSF dynamics and enhancing the interpretability of our findings. Fifth, the reproducibility of MRI scans themselves, alongside the reproducibility of reader measurements, is crucial for ensuring the reliability and applicability of our findings in clinical settings. This aspect is particularly important when considering the implementation of these techniques in routine clinical practice. Finally, these patients typically presented with immature skeletal maturity, coupled with progressive scoliosis. To ascertain whether aberrant CSF dynamics indeed instigate scoliosis, longitudinal studies are imperative.

Despite these limitations, our study provides valuable preliminary insights into the role of CSF dynamics in the progression of AIS and underscores the potential of advanced imaging techniques in elucidating this complex relationship. The findings pave the way for future research, advocating for the development of non-invasive methodologies and longitudinal studies that could overcome current limitations. Thus, our study is not only worthwhile but also a crucial addition to the existing literature, expanding our understanding of AIS and informing future clinical practices.

## Conclusion

In conclusion, this study revealed the intricate relationships between CSF dynamics and AIS, uncovering potential avenues for further research and highlighting the necessity for personalized approaches in AIS management and treatment. Comparative studies involving healthy controls could offer additional insights into the normal range of TD and its deviations in AIS.

### Supplementary Information

Supplementary Legends.

Supplementary Movie 1.

Supplementary Movie 2.

## Data Availability

The datasets used and/or analyzed during the current study available from the corresponding author on reasonable request.
