# Supplementary material for: The significance of cerebrospinal fluid dynamics in adolescent idiopathic scoliosis using time-SLIP MRI
Source: Sci Rep. 2024 May 28;14:12214. doi: 10.1038/s41598-024-63135-3 (PMC11133356; doi:10.1038/s41598-024-63135-3)
Supplement: Supplementary file 1 — Supplementary Legends. [file 41598_2024_63135_MOESM1_ESM.docx]

**Supplemental materials**

**Same patients as Figure4**

**Movie 1:** Time-SLIP MRI of 14-year-old Lenke type 1 patient.

**Movie 2:** Time-SLIP MRI of 19-year-old Lenke type 5 patient.
